# Aniline Is an Inducer, and Not a Precursor, for Indole Derivatives in *Rubrivivax benzoatilyticus* JA2

**DOI:** 10.1371/journal.pone.0087503

**Published:** 2014-02-12

**Authors:** Mujahid Mohammed, Sasikala Ch, Ramana V. Ch

**Affiliations:** 1 Department of Plant Sciences, School of Life Sciences, University of Hyderabad, Hyderabad, India; 2 Bacterial Discovery Laboratory, Centre for Environment, IST, J NT University Hyderabad, Kukatpally, Hyderabad, India; University Paris South, France

## Abstract

*Rubrivivax benzoatilyticus* JA2 and other anoxygenic photosynthetic bacteria produce indole derivatives when exposed to aniline, a xenobiotic compound. Though this phenomenon has been reported previously, the role of aniline in the production of indoles is still a biochemical riddle. The present study aims at understanding the specific role of aniline (as precursor or stimulator) in the production of indoles and elucidating the biochemical pathway of indoles in aniline-exposed cells by using stable isotope approaches. Metabolic profiling revealed tryptophan accumulation only in aniline exposed cells along with indole 3-acetic acid (IAA) and indole 3-aldehyde (IAld), the two major catabolites of tryptophan. Deuterium labelled aniline feeding studies revealed that aniline is not a precursor of indoles in strain JA2. Further, production of indoles only in aniline-exposed cells suggests that aniline is an indoles stimulator. In addition, production of indoles depended on the presence of a carbon source, and production enhanced when carbon sources were added to the culture. Isotope labelled fumarate feeding identified, fumarate as the precursor of indole, indicating *de novo* synthesis of indoles. Glyphosate (shikimate pathway inhibitor) inhibited the indoles production, accumulation of tryptophan, IAA and IAld indicating that indoles synthesis in strain JA2 occurs via the *de novo* shikimate pathway. The up-regulation of anthranilate synthase gene and induction of anthranilate synthase activity correlated well with tryptophan production in strain JA2. Induction of tryptophan aminotransferase and tryptophan 2-monooxygenase activities corroborated well with IAA levels, suggesting that tryptophan catabolism occurs simultaneously in aniline exposed cells. Our study demonstrates that aniline (stress) stimulates tryptophan/indoles synthesis via the shikimate pathway by possibly modulating the metabolic pathway.

## Introduction

Aromatic compounds constitute the second most abundant class of organic compounds and are natural or anthropogenic. Since the industrial revolution, diverse array of aromatic chemicals has been synthesized and due to their extensive use, are continuously released into the environment [1], [2]. Aromatic compounds are one of the major groups of xenobiotic compounds [3], [4]. Many of these chemicals are persistent, toxic to life forms [2], and act as stressors [5], [6]. Bacteria overcome these deleterious effects by employing various strategies, mainly active degradation/detoxification of these compounds. Some bacteria degrade these aromatic compounds for growth [4] and others transform these compounds to less toxic forms (detoxification) [7], [8].

One such aromatic compound is aniline, a primary aromatic amine widely used in manufacturing of dyes, plastics, rubber additives, paints, herbicides, pesticides and in pharmaceutical industries [9]. Aniline is also formed by microbial transformation of nitroaromatic compounds and aniline based pesticides [10]. Owing to its extensive usage, large quantities of aniline is released into the environment by industrial effluents [9] and is accumulated in sediments, sludge and agricultural soil [10]. Aniline is considered as one of the important pollutants due to its recalcitrant properties [EEC, 1976; Federal Register, 1979) and is on the priority pollutant list of US Environmental Protection Agency [Federal Register, 1979). Aniline and its derivatives are known to be mutagenic, and their carcinogenicity has been reported in higher animals and mutagenecity in bacteria [11], hence its fate in the environment is of serious concern.

Metabolism of aniline and its derivatives by soil microorganisms was considered to be an effective measure for bioremediation [12]. Many aniline degrading bacteria were isolated from agricultural soils and industrial areas [9], [10]; aerobic and anaerobic degradation pathways of aniline were also studied [4], [9], [13]. Biotransformation of aniline by soil microorganisms is one of the strategies of aniline detoxification [14], [15]. Aniline and its derivatives were transformed to less toxic acetanilides by arylamine N-acetyltransfarase (NAT). NAT detoxifies aniline xenobiotics by transferring the acetyl group from acetyl-CoA to the nitrogen atom of arylamine [15]. Aniline transformation to formanilide by N-formylation and to aminophenol by hydroxylation was observed in some bacteria [16]. Transformation of aniline to anthranilate (a precursor of tryptophan) was reported in bacteria. Aniline was observed as major catabolite of diphenylamine, nitrodiphenylamine, and the formation of indole was also observed; the authors speculated that aniline was a possible precursor of indole [17].

Production of indole esters in the presence of aniline was also reported in a purple bacterium, *Rhodobacter sphaeroides* OU5, which, however, was dependent on the presence of a carbon substrate such as fumarate [18]. Similarly, production of tryptophan and other indole derivatives in the presence of aniline was reported in other purple bacteria [19] and these studies led to the speculation that aniline might be a precursor or an inducer for indole biosynthesis. However, this biochemical enigma has not been resolved so far. To address this question, we have employed stable isotopic and biochemical approaches to decipher the biosynthesis of indoles in aniline-exposed cells of a photosynthetic bacterium, *Rubrivivax benzoatilyticus* JA2. Our study demonstrated that aniline is not a precursor for indole biosynthesis; rather, it induces indole biosynthesis in strain JA2. Our results also suggest a possible stress-induced metabolic re-programming and shift towards synthesis of indoles in the presence of aniline.

## Materials and Methods

### Bacterial strain and culture conditions

*Rubrivivax benzoatilyticus* JA2^T^ (ATCC BBA-35) strain was used in this study. Strain JA2 was grown photoheterotrophically (anaerobic, 30°C; light 2,400 lux) in a mineral medium [20] supplemented with malate (22 mM) as the carbon source and ammonium chloride (7 mM) as the nitrogen source in fully filled screw cap test tubes (10×100 mm) or reagent bottles (250 ml) at pH 6.8, 30±1°C. Photoheterotrophically grown late-log phase culture (0.45 OD_660 nm_) of *R. benzoatilyticus* JA2 was used for all the experiments.

Effect of aniline on growth of strain JA2 was monitored by supplementing different concentrations of aniline (filter sterilized) to media and incubated under light at 30°C. Late log phase (OD_660 nm_ 0.4) culture was exposed to aniline (25 mM) and fumarate (13 mM) while the control received only fumarate. Aniline and control cultures were incubated under photoheterotrophic conditions for desired period (24 or 48 h); then cells were harvested and supernatant was used for total indoles estimation and extraction of metabolites. For studies with resting cell suspensions, late log phase culture (OD_660 nm_ 0.4) was harvested by centrifugation (10,000×g, 4°C, 10 min), washed twice with mineral medium (devoid of carbon and nitrogen sources) and finally suspended in the same mineral medium and exposed to aniline as described previously.

Effect of glyphosate on growth of strain JA2 during exposure to aniline/anthranilate was monitored by supplementing different concentrations of glyphosate (filter sterilized) to media. Growth restoration of strain JA2 (in presence of glyphosate) was performed by amending 1 mM of glyphosate to culture, after 20 h of incubation, the culture was supplemented with aromatic amino acids (150 µM of phenylalanine/tyrosine) or anthranilate (150 µM). Effect of glyphosate on indole production was monitored by supplementing 1 mM of glyphosate to culture during aniline/anthranilate exposure. Indoles were estimated by Salper's reagent using indole as standard [21].

### Stable isotope precursor feeding

Late log phase (OD_660 nm_ 0.4) cultures of strain JA2 were supplemented with deuterium labeled aniline (Aniline-d_5_, 98 atom %D Sigma Aldrich) or fumarate (Fumaric acid-d_4_, 98 atom % D). After 48 h of incubation, cultures were harvested and supernatant was used for LCMS analysis.

### Extraction of metabolites and HPLC analysis

Cultures were harvested (10,000×g, 4°C, 10 min) and supernatant was concentrated to dryness under vacuum in rotary flash evaporator (Heidolph, Germany) at 45°C. After complete dryness, the brown residue obtained was fractionated according to the protocol used by Powell [22]. Indolic fractions were evaporated to dryness using flash evaporator, redissolved in HPLC grade methanol (1 ml), filtered (0.22 µm membrane, SUPRO Millipore) and used for HPLC and mass spectrometric analysis. HPLC analysis was performed according to Mujahid *et al* [19]. HPLC was performed on Prominence LC20AT system (Shimadzu-Japan) equipped with diode array detector and Phenomenex C-18 column (Luna 5 µm, 250×4.6 mm). Metabolites were separated by using a linear gradient programme of 30 min (Solvent A; 1% acetic acid, solvent B; 100% acetonitrile) with a flow rate of 1.5 ml/min. Injection volume was 20 µl and metabolites were detected at 280, 325 nm. The metabolites, aniline (R_t_; 5.4 min), tryptophan (9.5 min), anthranilate (14.5 min), indole-3-aldehyde (18.5 min) and indole-3-acetic acid (19.1 min) were quantified with reference to the peak areas of known concentrations of authentic standards. Fumarate was measured directly from culture supernatants by using the same HPLC method (as described above) except that fumarate was monitored at 230 nm. Culture supernatant was filtered (0.22 µm membrane, SUPRO Millipore), 20 µl of filtrate was injected into HPLC and fumarate (R_t_ 2.45 min) was quantified with reference to peak areas of known concentrations of authentic standard.

### ESI-LC-MS/MS analysis

Mass analysis was performed according to Mujahid *et al* [19] on MicroTOF-Q (Brukarsdeltanoics) mass spectrometer coupled to HPLC (Agilent 1200 series) with UV-VIS detector. Metabolites were separated on Waters C-18 (5 µm, 150×4.6 mm) column. HPLC conditions were as same as described previously, except that the flow rate was 0.8 ml/min and the injection volume was 10 µl. Samples were introduced via auto sampler and metabolites were monitored at 280 nm. With a post column split effluent was introduced into electro spray ionization (ESI) ion source (80°C, Cone voltage 15–25V). ESI (+) and ESI (−) ion modes were used to detect molecular ion masses ([M]^+^) and collision energy of 5–20 eV was used for fragmentation depending upon the nature of the molecule. Mass spectra were recorded from 50–1000 Da.

### Preparation of cell free extracts and enzyme assays

Control or aniline exposed cells of *R. benzoatilyticus* JA2 were used for preparation of cell free extracts. Cells were harvested by centrifugation (4°C, 10,000×g, 10 min), the cell pellet was washed twice with Tris-HCl buffer (50 mM, pH 7.8) and suspended in 4 ml of same buffer containing 50 µM PMSF (phenylmethylsulfonyl fluoride). Cells were lysed by sonication with MS-70 probe (Bandelin, German, model-UW 2070) at 45% power, 4°C, 7 cycles of 1 min duration and 5 min gap between each cycle. After sonication, lysate was centrifuged (20,000×g, 20 min, 4°C) and the clear supernatant obtained was used as the source of enzyme. Protein content was measured by Bradford's method [23] using bovine serum albumin as standard.

For the assay of anthranilate synthase activity, the reaction mixture contained 1 mM chorismate, 1 mM glutamine, 50 µM MgCl_2_ and cell free extract (1.5 ml) in a final volume of 3 ml. After 30 min of incubation at 37°C, the reaction was stopped by adding 5N HCl (300 µl). Reaction mixture was centrifuged and product (anthranilate) was extracted from the supernatant by ethyl acetate. Ethyl acetate was concentrated and dissolved in methanol and analysed by HPLC as described above. One unit (U) of enzyme activity was defined as the amount of enzyme required for the formation of 1 µmole of product. Specific activity was expressed as unit activity per mg protein.

Assay of tryptophan aminotransferase enzyme activity was carried out in a final volume of 3 ml containing L-tryptophan (1 mM), α-ketoglutarate (1 mM), 50 µM of PLP (pyridoxal-5′-phosphate) as cofactor and cell free extract (ml) [24]. After 20 min incubation at 37°C, the reaction was stopped by adding 300 µl of 5N HCl. Reaction mixture was centrifuged (4°C for 10 min at 10,000 g) and the supernatant was collected and extracted twice with ethyl acetate. The ethyl acetate layers were pooled, evaporated to dryness under vacuum, dissolved in 100 µl methanol and the product IAA (indolepyruvate is unstable and readily converts into IAA) was analysed by HPLC. Assay of tryptophan 2- monooxygenase enzyme activity was carried [24] out in a final volume of 0.7 ml Tris buffer (50 mM, pH 7.8) containing 0.5 mM of L-tryptophan and appropriate amount of cell free extract (300 µl). Reactions were incubated at 37°C for 20 min and then reaction was stopped by adding 100 µl of 5N HCl. Reaction mixture was centrifuged (4°C for 10 min at 10,000×g) and the supernatant was collected and extracted twice with ethyl acetate. The ethyl acetate layers were pooled, evaporated to dryness under vacuum, dissolved in 50 µl of methanol and the product (indole-3-acetamide) was analysed by HPLC. One unit (U) of enzyme activity was defined as the amount of enzyme required for the formation of 1 µmole (tryptophan aminotransferase) or 1 nmole (tryptophan monooxygenase) of product. Specific activity was expressed as unit activity per mg protein. Pre-denatured enzyme (denatured by adding 300 µl of 5N HCl) was taken as blank.

### RNA preparation and quantitative real time RT-PCR

For RNA isolation all the plastic ware was treated with 0.1% (v/v) DEPC (Diethylpyrocarbonate) overnight, dried and autoclaved. *R. benzoatilyticus* JA2 was grown photoheterotrophically (0.45 OD_660 nm_) and exposed to aniline as described. 2.5 ml culture was harvested at 60 and 140 min after the aniline exposure, flash frozen in liquid nitrogen and stored at −80°C. RNA was isolated by RNase mini kit (Qiagen) according to manufacturer's instructions. Before RNA isolation, cells were treated with lysozyme (30 mg/ml in Tris.HCl buffer pH 7.0) for 5 min. on column. DNase treatment was performed with DNase I (Qiagen) according to manufacturer's instructions. Isolated RNA was stored at −80°C until further analysis. RNA integrity was analysed on Bioanalyser 2100 and quantity by Nanodrop (Thermo Scientific). DNase treated RNA (925 ng) was reverse transcribed to make 37 ng/µl of cDNA using the Affinity Script QPCR cDNA synthesis kit (Agilent - Lot# 6077352) according to manufacturer's protocol. Real time PCR was performed by using Brilliant II SYBR Green qPCR Master mix (Lot # 1105284). Primers used in real time PCR analysis are listed in Table S1. Each sample was run in duplicates for each gene using 37 ng input per reaction and PCR conditions are as follows; initial denaturation at 95°C for 10 min followed by 40 cycles of 95°C for 30 s, 58°C for 1 min, 72°C for 1 min. A melt curve was also performed after the assay to check for specificity of the reaction. Real time PCR was performed on Stratagene Mx3005P (Agilent technologies) platform. The relative expression levels of the genes were determined after normalizing with 16S rRNA (reference gene) by using Delta Ct method [25].

## Results

### Time course of aniline and fumarate utilization and indoles production

**Figure 1:**
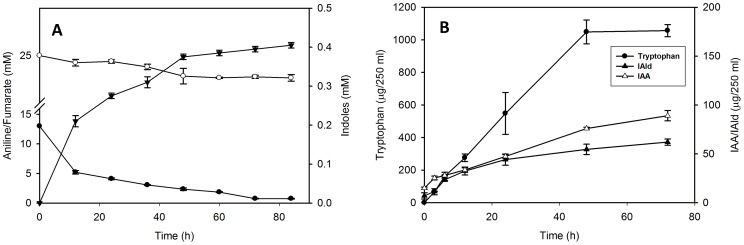
Time course production of indoles by strain JA2. Time course analysis of aniline (Open circles], fumarate (solid circles] utilization and indoles (triangles] production by *R. benzoatilyticus* JA2 (A). Data represents mean ± standard deviation of three independent experiments done in duplicates. Time course analysis of Trp, IAA and IAld production in aniline exposed cells (B). Trp, tryptophan; IAA, indole-3-acetic acid; IAld, indole-3-aldehyde. Data represents mean ± standard deviation of three independent experiments.

Utilization of aniline and fumarate was observed with concomitant production of indoles (Figure 1A). After 48 h of aniline exposure 0.35–0.39 mM indoles were produced while 0.4–0.45 mM of aniline was utilized. Thus yields of indoles were nearly equal to that of aniline utilized.

### Tryptophan, IAA, and IAld produced by aniline supplemented culture

Tryptophan, indole 3-acetic acid (IAA), and indole 3-aldehyde (IAld) were identified as major indole metabolites in aniline supplemented cultures of *R. benzoatilyticus* JA2. When tryptophan, IAA, and IAld levels were monitored, tryptophan accumulation (759±25 µg/250 ml) was observed only in aniline supplemented cultures while no detectable amount of L-tryptophan was observed in control (Figure S1A). IAA and IAld were detected both in control and aniline supplemented cultures, however, IAA and IAld levels were significantly high (IAA 175±30 µg/250 ml; IAld 96±25 µg/250 ml) in aniline supplemented culture compared to that of control (IAA 10±3 µg/250 ml; IAld 8±2.5 µg/250 ml) (Figure S1A). Time course analysis of tryptophan, IAA, and IAld was performed and no detectable amount of tryptophan was found at zero minutes; tryptophan levels steadily increased from 3 h and reached maximum (1048.5±72 µg/250 ml) at 48 h (Figure 1B) of aniline supplementation. Similarly, IAA and IAld levels increased from 3 h and reached a maximum (IAA 89±5 µg/250 ml; IAld 62±3 µg/250 ml) at 72 h (Figure 1B). Tryptophan levels were significantly high (1048.5±72 µg/250 ml) compared to that of IAA (89±5 µg/250 ml) and IAld (IAld 62±3 µg/250 ml) and rate of tryptophan accumulation was also high (Figure 1B). Tryptophan production increased with increasing concentration of aniline and tryptophan/indoles production was high at 25 mM of aniline (Figure S1B).

### Probing indoles biosynthesis by aniline-d_5_ stable isotope feeding reveals that aniline is not a precursor of indoles

To evaluate the role of aniline, cells were cultured with deuterium labeled and unlabeled aniline; indoles were extracted from supernatant and analyzed by mass spectrometry. If aniline acts as a precursor of indoles, molecular mass of indole would increase by 4 mass units (+4 a.m.u.as aromatic ring retains four deuterium atoms; Figure S2A). Increase of four mass units was not observed in mass spectrum of tryptophan from labeled aniline fed culture (molecular ion mass of tryptophan, 205.10 [M+H]; Figure S2B). Similarly, four mass units increase was not observed in the case of either IAA (176.06 [M+H]) or IAld (145.05 [M+H]; Figure S3). This result indicated that aniline was not a precursor of indoles (tryptophan, IAA, IAld).

### Indoles are synthesized via the shikimate pathway in aniline exposed cells

**Figure 2:**
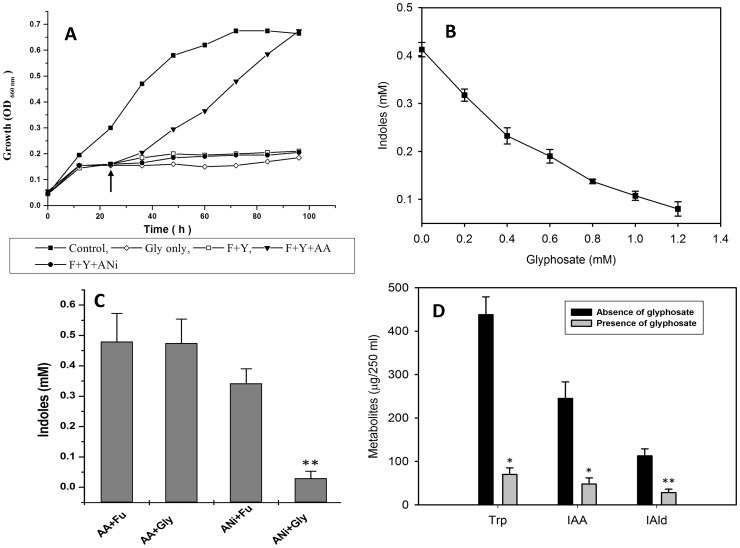
Effect of glyphosate on growth and indoles production in strain JA2. Inhibition of growth of *R. benzoatilyticus* JA2 by glyphosate and restoration of growth by aromatic amino acids (A). Culture was grown on malate media with glyphosate (1 mM] for 22 h. Arrow indicate supplementation (150 µM] of phenylalanine, tyrosine, anthranilate or aniline (10 mM]. F, phenylalanine; Y, tyrosine; AA, anthranilate; ANi, aniline; Gly, glyphosate. Effect of glyphosate on indoles production in aniline supplemented cultures of *R. benzoatilyticus* JA2 (B). Data represents mean ± standard deviation of three independent experiments done in duplicates. Effect of glyphosate on indoles production by anthranilate (AA] and aniline (ANi] supplemented cultures of *R. benzoatilyticus* JA2 (C). Data represents mean ± standard deviation of three independent experiments done in duplicates. Fu, fumarate; **, p value<0.005 compared with glyphosate un-supplemented cultures. Effect of glyphosate on Trp, IAA and IAld production by aniline exposed cultures of *R. benzoatilyticus* JA2 (D). Data represents mean ± standard deviation of three independent experiments. *, p value<0.05; **, p<0.005 compared with glyphosate un-supplemented cultures obtained from Student's *t*test.

Aromatic metabolites (including tryptophan/indoles) are biosynthesized via the shikimate pathway in bacteria and plants [26]. The role of the shikimate pathway in indoles production can be ascertained by using glyphosate, an inhibitor of the enzyme enoylpyruvyl shikimate phosphate synthase from the shikimate pathway [26]. When glyphosate (1 mM) was added to the media, growth of *R. benzoatilyticus* JA2 was inhibited as the cells were starved of aromatic amino acids. When the culture was then supplemented with aromatic amino acids (150 µM) growth was completely restored (data not shown) indicating that shikimate pathway was operative in *R. benzoatilyticus* for aromatic aminoacid synthesis since glyphosate specifically inhibits the shikimate pathway blocking aromatic amino acid synthesis/production. Aniline is a structural analogue of anthranilate (tryptophan precursor). We tested whether aniline could act as precursor for tryptophan biosynthesis. Growth inhibition of *R. benzoatilyticus* JA2 (Figure 2A, for 22 hrs) could be resumed and was completely restored when the culture was supplemented with phenylalanine, tyrosine, and anthranilate. However, when anthranilate was replaced with aniline, growth did not resume (Figure 2A), confirming that aniline is not a precursor of tryptophan synthesis. Furthermore, glyphosate inhibited the production of indoles by aniline supplemented cultures in a dose dependent manner (Figure 2B). Strain JA2 produces indoles when supplemented with anthranilate, and when glyphosate was added to the anthranilate supplemented culture, production of indoles was not inhibited, whereas in aniline supplemented cultures production was inhibited (Figure 2C). These results indicate that aniline is not a precursor of indoles, rather indoles are *de novo* synthesized by the shikimate pathway in the presence of aniline. Tryptophan, IAA, and IAld production was significantly inhibited when glyphosate was added to the culture compared to that of no glyphosate (Figure 2D). These results unequivocally demonstrate that indoles were biosynthesized via the shikimate pathway in aniline supplemented cultures.

### Indoles production by aniline exposed cells is dependent on carbon source availability

Supplementation of carbon sources to aniline exposed cells of *Rhodobacter sphaeroides* OU5 enhanced the production of indoles [27]. Similarly, production of indoles was high (0.34±0.06 mM (fumarate), 0.28±0.04 mM (pyruvate), 0.25±0.05 mM (malate) when carbon sources were supplemented to the culture compared to without supplementation (0.012±0.04 mM). Further, when both pyruvate and fumarate were supplemented to aniline exposed cells, indoles production significantly increased (0.41±0.06 mM). Moreover, when a non-metabolisable carbon source such as maleate was supplemented to aniline exposed cells, indole production was significantly decreased (0.07 mM) and these results indicate that metabolizable carbon sources enhance indole production.

**Figure 3:**
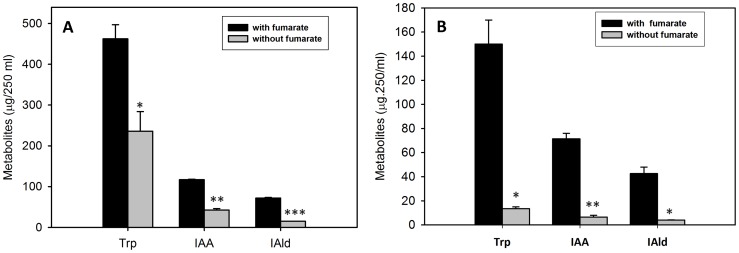
Effect of fumarate supplementation on indoles production by aniline exposed cultures of *R. benzoatilyticus* JA2. Production of indoles by growing cells (A), and by resting cell suspensions (B). Data represents mean ± standard deviation of three independent experiments. *, p value<0.05; **, p<0.005; ***, p<0.0005 compared with fumarate unsupplemented cultures obtained from Student's *t* test.

When strain JA2 was exposed to aniline with and without supplementation of fumarate (to understand the role of fumarate in indoles production), tryptophan (462 µg.250 ml^−1^), IAA (117 µg.250 ml^−1^), and IAld (72 µg.250 ml^−1^) levels were high in fumarate supplemented cultures while in absence of fumarate, tryptophan (236 µg.250 ml^−1^), IAA (42.5 µg.250 ml^−1^), and IAld (15 µg.250 ml^−1^) levels were low (Figure 3A). Further, when similar experiments were performed with resting cell suspensions, tryptophan, IAA, and IAld production was significantly high in fumarate supplemented cultures compared to cultures without fumarate supplementation (Figure 3B). These results suggest that fumarate is a possible precursor of indole metabolites.

### Fumarate is the precursor of indole metabolites

**Figure 4:**
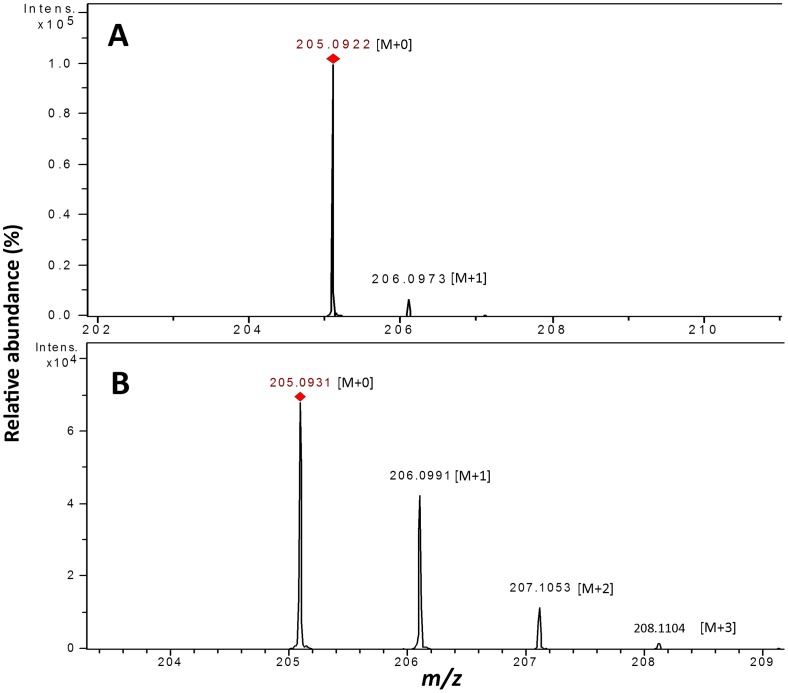
Stable isotope labeled fumarate feeding of aniline exposed cultures of *R. benzoatilyticus* JA2. Mass spectrum of tryptophan from unlabeled fumarate supplemented culture (A) and from labeled fumarate supplemented culture (B). M, molecular ion mass; 0,1,2,3 denote number of deuterium atoms incorporated.

**Figure 5:**
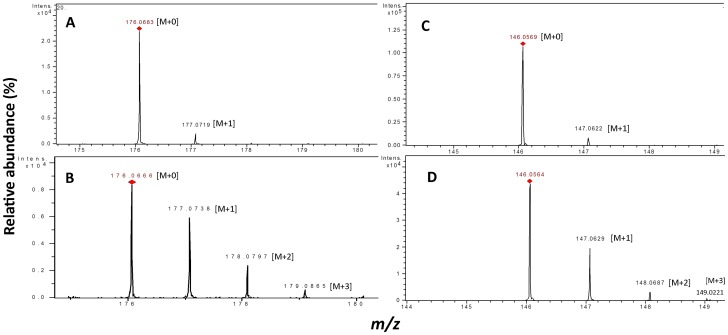
Stable isotope labeled fumarate feeding to probe IAA and IAld synthesis in *R. benzoatilyticus* JA2. Mass spectrum of IAA from unlabeled fraction (A), labeled fraction (B), mass spectrum of IAld from unlabeled fraction (C) and labeled fraction (D). M, molecular ion mass; 0,1,2,3 denote number of deuterium atoms incorporated.

To confirm whether fumarate is a precursor of indoles or not, deuterium labeled (fumarate-d_4_) or unlabeled fumarate was supplemented to strain JA2 during aniline exposure, and indole metabolites were extracted and subjected to mass analysis. Mass analysis of tryptophan from labeled fumarate fed cultures revealed molecular ion masses of 205[M+0]^+^, 206[M+1]^+^, 207[M+2]^+^ and 208[M+3]^+^ indicating the incorporation 1, 2, 3 of deuterium atoms, respectively (Figure 4B). Tryptophan mass spectrum from unlabeled fumarate fed cultures revealed molecular ion masses of 205[M+0] and 206[M+1]^+^ only (Figure 4A). Similarly, mass spectrum of IAA and IAld from labeled fumarate fed culture revealed molecular ion mass of 176[M+0]^+^, 177[M+1]^+^, 178[M+2]^+^,179[M+3]^+^ (Fig. 5B) and 146[M+0]^+^, 147[M+1]^+^, 148[M+2]^+^, 149[M+3]^+^, respectively (Fig. 5D). Mass spectrum of IAA and IAld from unlabeled fumarate fed cultures revealed molecular ion masses 176[M+0]^+^, 177[M+1]^+^ (Figure 5A) and 146[M+0]^+^, 147[M+1]^+^ (Figure 5C) only. Relative abundance of isotopologues of all three metabolites was significantly high in labelled fractions compared to unlabeled (Figure S4A). Molecular ion masses of M+1, M+2, M+3 and M+4 (trace amount <1%) of tryptophan, IAA, and IAld suggest incorporation of deuterium atoms (1 to 4) into these metabolites (Table S2). The mass fragmentation pattern of tryptophan resulted in a fragment of 146 [m/z]; this fragment corresponds to the open indole ring structure (Figure S4B), and the increase of molecular ion mass (1 to 4 mass units) of this fragment from labeled fumarate fed fraction (Table S2) possibly suggests that incorporated deuterium atoms are on indole molecule. Similarly, IAA and IAld mass fragmentation indicated a major molecular ion mass of 130 [m/z] and 118 [m/z], respectively; these fragments correspond to intact indole molecules (Figure S4B) and an increase of molecular ion mass (1 to 4 units) of these fragments from labeled fractions (Table. S2) suggest that deuterium atoms to be on indole molecule. These results strongly confirm that fumarate is precursor of indoles (tryptophan, IAA, and IAld) synthesis in *R. benzoatilyticus* JA2.

### Induction of anthranilate synthase activity correlates with indoles production in aniline supplemented cultures

Anthranilate synthase participates in the first committed step of tryptophan biosynthesis and it is a rate limiting step [26]. Anthranilate synthase activity was monitored and specific activity of anthranilate synthase was significantly high (78.5 U mg protein^−1^) in aniline exposed cultures compared to that of unexposed culture (24.7 U mg protein^−1^). Correlation between induction of anthranilate synthase activity and tryptophan/indoles accumulation indicate its role in indoles synthesis.

### Real time PCR (qRT-PCR) analysis of shikimate pathway genes reveals up-regulation of anthranilate synthase gene but not other genes

**Figure 6:**
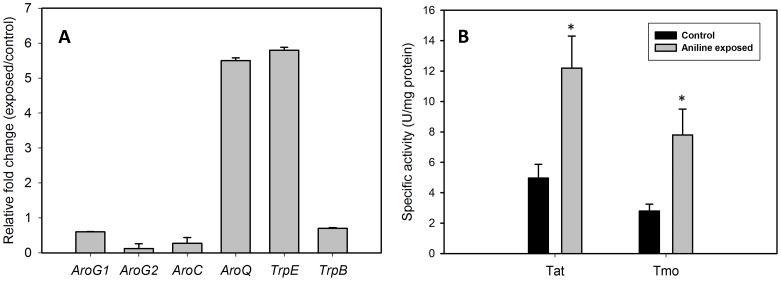
Transcript levels of shikimate, tryptophan biosynthetic genes and tryptophan catabolising enzyme activities. Real time quantitative PCR analysis of shikimate and tryptophan biosynthetic genes in *R. benzoatilyticus* JA2 (A). Data represents mean standard deviation of two independent experiments with two biological replicates. *AroG1/AroG2* corresponds todeoxy-D-arabinoheptulosonate 7-phosphate synthase; *AroC*, chorismate synthase; *AroQ*, chorismatemutase; *TrpE*, anthranilate synthase; *TrpB*, tryptophan synthase β. Tryptophan catabolizing enzyme activities of control and aniline exposed cultures of *R. benzoatilyticus* JA2 (B). Tat, tryptophan aminotransferase; Tmo, tryptophan 2-monooxygenase. Data represents mean standard deviation of three independent experiments. *, p<0.05 compared with control exposed cells obtained from Student's *t* test.

Real time PCR analysis of 3-deoxy-D-arabinoheptulosonate 7-phosphate (DAHP) synthase (*AroG*), chorismate synthase (*AroC*), chorismatemutase (*AroQ*), anthranilate synthase (*TrpE*), tryptophan synthase-β subunit (*TrpB*) genes was performed to check the transcript levels of these genes in aniline exposed and unexposed cultures. Significant changes in the transcript levels of selected genes was not observed at 20 min of aniline exposure. Chorismate mutase (*AroQ*) (5.5 fold) and anthranilate synthase (*TrpE*) (5.6 fold) genes were up-regulated in aniline exposed cells compared to control after 60 minutes of aniline exposure (Figure 6A). Significant change was not observed in expression of DAHP synthase (*AroG1and G2*), chorismate synthase (*AroC*) or tryptophan synthase-β genes (*TrpB*).

### Induction of tryptophan aminotransferase (TAT) and tryptophan 2-monooxygenase (TMO) activities correlates with IAA accumulation

IAA and IAld were two major indole metabolites identified in aniline exposed cultures and IAA synthesis from tryptophan was reported in strain JA2 previously [24]. Tryptophan aminotransferase (TAT) and tryptophan 2-monooxygenase (TMO) participate in first step of tryptophan catabolism leading to the formation of IAA. Assay of enzyme activities of TAT and TMO were performed and specific activities of tryptophan aminotransferase (12.2 U mg protein^−1^) and monooxygenase (7.8 U mg protein^−1^) were significantly high in aniline exposed cultures compared to that of unexposed (aminotransferase, 4.9 U; monooxygenase, 2.8 U mg protein^−1^) (Figure 6B).

## Discussion

Bacteria are constantly exposed to biotic and abiotic cues, and one such cue is aromatic compounds. One such aromatic compound, aniline, is degraded by different groups of bacteria. Transformation of aniline to anthranilate was reported in *Rhodococcus* and possible transformation of aniline to indole is reported elsewhere in bacteria [17]. Indole metabolites were observed in aniline exposed cultures of few purple bacteria [19]. Accumulation of indole derivatives in aniline supplemented cultures and reports of aniline transformation to anthranilate, a precursor of indole/tryptophan, suggested that aniline may be a precursor of indoles. Surprisingly, in the present study with stable isotope labeled aniline feeding, inhibition of indoles production by glyphosate (Figure 2B) indicated that aniline is not a precursor of tryptophan/indoles. Though aniline is not used as carbon source or as precursor of indoles by strain JA2, uptake of aniline (0.45 mM) was observed and this suggests a possible transformation of aniline to some metabolites which are yet be identified. However, aniline is required to induce indoles production by an unknown mechanism and this may be a stress response to aniline. Stress induced indoles accumulation is reported in bacteria. For example, stresses like antimicrobial [28]–[30], xenobiotic [17], [31], nutritional (carbon, nitrogen starvation)/environmental (pH, temperature, growth phase of culture) [32] and oxidative stress [28]–[30] stimulated indoles synthesis. Ethanol (stress) induced tryptophan accumulation is reported in yeast [33] and in plants wide a variety of abiotic [34], [35] or biotic cues trigger accumulation of tryptophan and its derivatives [36]–[38]. Acifluorfen (herbicide) triggered synthesis of an indolic compound (camalexin) in *Arabidopsis thaliana* [39] and methanol triggered accumulation of tryptophan/its derivatives in rice [34]. Diverse group of bacteria produce indole metabolites and production of indoles is reported to have fitness benefit to bacteria under different physiological conditions [40]–[42]. However, role of *de novo* pathway of indoles and its regulation under stress is not understood well in bacteria.

Indoles accumulation in this study was dependent on carbon source availability (Figure 3A, B) and this plausibly suggests *de novo* synthesis. Many aromatic compounds including indoles are *de novo* synthesised via the shikimate pathway in bacteria and plants [43]. In this study, when *de novo* synthesis was blocked by glyphosate (shikimate pathway inhibitor), indoles production was inhibited (Figure 2B); moreover, inhibition was observed only in aniline exposed cells but not in anthranilate (Figure 2C) or tryptophan (data not shown) supplemented cultures. This is presumably because glyphosate inhibits 5-enolpyruvylshikimate 3-phosphate synthase, the penultimate enzyme of chorismate synthesis [44], which is required for tryptophan/indoles biosynthesis, thus strongly suggesting that indoles are biosynthesised via the shikimate pathway in aniline exposed cells. Similarly, glyphosate inhibited the stress (oxidative) induced accumulation of tryptophan and its derivative (camalexin) in *Arabidopsis* [39] and tryptamine accumulation in rice infected with *Magnaporthegrisea*, which were known to synthesise indoles via shikimate pathway [45]. However, *de novo* synthesis requires a plausible flux of carbon and energy towards tryptophan/indoles synthesis. In support of this we found enhanced production of tryptophan, IAA and IAld in fumarate supplemented conditions (Figure 3A,B) and this suggests the possible role of fumarate as a carbon and energy source. Furthermore, fumarate stable isotope precursor feeding studies (Figure 4 and 5) confirmed fumarate as a precursor of indoles; this result strongly suggests a flux of carbon (fumarate) to tryptophan biosynthesis. Aromatic compounds synthesis is a complex metabolic process and availability of precursors, erythrose-4-phosphate (E4P) and phosphoenolpyruvate (PEP) are critical for their production [46]. Proteome analysis of aniline exposed cells revealed up-regulation (unpublished data) of two key enzymes, transketolase (tkt) and phosphoenolpyruvate carboxykinase (pck), involved in synthesis of E4P, PEP and this may result in increased pool of precursors for Trp synthesis. Over expression of *tkt* and *pck* genes in bacteria enhanced the aromatic compounds/tryptophan production by providing precursors (E4P, PEP) [43], [47]. Up-regulation of tkt and pck genes possibly suggests a flux of fumarate to the pentose phosphate pathway and gluconeogenesis. Carbon flow from the shikimate pathway towards tryptophan biosynthesis is regulated by anthranilate synthase (TrpE), a key enzyme of tryptophan biosynthesis, and is subject to transcriptional and feedback regulation by tryptophan [48]. In this study, increased anthranilate synthase activity and up-regulation of the anthranilate synthase gene (Figure 6A) corroborated well with the accumulation of tryptophan/indoles in aniline exposed cells. Over expression of the *TrpE* gene resulted in enhanced production of tryptophan/indoles in bacteria [49]. Up-regulation of anthranilate synthase gene and tryptophan accumulation were observed during biofilm formation of *Salmonella entericasvtyphimurium* [33] and *E. coli*. Similarly in *Saccharomyces cerevisiae* ethanol stress stimulated tryptophan accumulation [41]. Though transcript levels of DAHP synthase, chorismate synthase, and tryptophan synthase genes were not changed significantly in *R. benzoatilyticus* JA2 (Figure 6A), accumulation of tryptophan was observed and this suggests that basal level expression of these genes may be sufficient for tryptophan synthesis. In the present study accumulation of IAA and IAld along with tryptophan suggests tryptophan catabolism in aniline exposed cells. This was further supported by increased tryptophan aminotransferase and tryptophan 2-monooxygenase activities (Figure 6B) involved in Trp catabolism. Tryptophan dependent IAA biosynthesis is predominant in bacteria [42] and two tryptophan dependent IAA biosynthetic pathways were reported in strain JA2 [24]. On the other hand IAld synthesis is not well understood and oxidation (non-enzymatic/non-specific oxidases) of IAA and indole-3-pyruvate is thought to be involved in IAld synthesis [50]. Nevertheless, tryptophan dependent biosynthesis of IAld was reported by our group in strain JA2 [24].

Interestingly, aniline stress induces accumulation of tryptophan and its derivatives in spite of complex biosynthetic and regulatory mechanisms of Trp synthesis in bacteria [48], [51]. However, organization of Trp genes and their regulation vary greatly in bacteria and this is governed largely by life style/selective pressure under which the bacteria thrive [51], [52]. Beyond being a precursor for proteins, tryptophan also serves other functions such as a precursor for pigments, antibiotics, and secondary metabolites at different physiological/developmental stages and this demands different regulatory mechanisms other than sensing tryptophan for protein synthesis [51], [52]. Due to the fact that tryptophan accumulated only in aniline exposed cells (but not in control), we speculate a possible decoupling of tryptophan synthesis (from sensing for protein synthesis) or a different kind of regulatory mechanism enabling cells to overproduce indoles on physiological demand. Biosynthesis of aromatic metabolites and its regulation has not previously been studied in *R. benzoatilyticus* JA2, and gaining insights into these mechanisms is of particular interest because it overproduces indoles. Our study demonstrates the role of *de novo* pathways in indoles production and also demonstrates a metabolic re-programming and shift towards tryptophan/indoles biosynthesis in response to xenobiotic (aniline) stress.

## Supporting Information

Figure S1 Tryptophan, IAA and IAld levels in control and aniline exposed cultures of *R. benzoatilyticus* JA2 (A). Tryptophan levels at different concentrations of aniline (B). Metabolites were quantified by HPLC and data represents mean standard deviation of three independent experiments.
(TIF)

Figure S2 Schematic representation of stable isotope labelled aniline precursor feeding experiments with *R. benzoatilyticus* JA2 (A). M, denotes molecular ion mass and 0, 4 number of deuterium atoms incorporation. Mass spectrum of tryptophan from unlabeled fraction (B) and mass spectrum from labeled fraction (C).
(TIF)

Figure S3 Mass spectrum of IAA from unlabeled (**A**) fraction and labeled fraction (**B**). Mass spectrum of IAld from unlabeled fraction (**C**) and from labeled fraction (**D**).
(TIF)

Figure S4 **Relative abundance of isotopologues of indoles metabolites from unlabeled and labeled fractions.** Area of each isotopologue obtained from mass analysis was used for the analysis (A). Predicted mass fragmentation of tryptophan, IAA and IAld according to Metlin data base (www.metlin.scripps.edu] (B).
(TIF)

Table S1 **Primers used for real time PCR analysis.**
(DOC)

Table S2 **Mass spectral analysis of Trp, IAA and IAld from stable isotope labeled fumarate fed cultures.** M denotes molecular ion mass; 1,2,3,4 denote number of deuterium atoms incorporated. NF, no fragmentation was obtained as they were in trace amount (<1% abundance). Molecular ion masses in bold indicate no deuterium atoms incorporation.
(DOC)
