# Supplementary figures and images for: Aniline Is an Inducer, and Not a Precursor, for Indole Derivatives in Rubrivivax benzoatilyticus JA2
Source: PLoS One. 2014 Feb 12;9(2):e87503. doi: 10.1371/journal.pone.0087503 (PMC3922755; doi:10.1371/journal.pone.0087503)

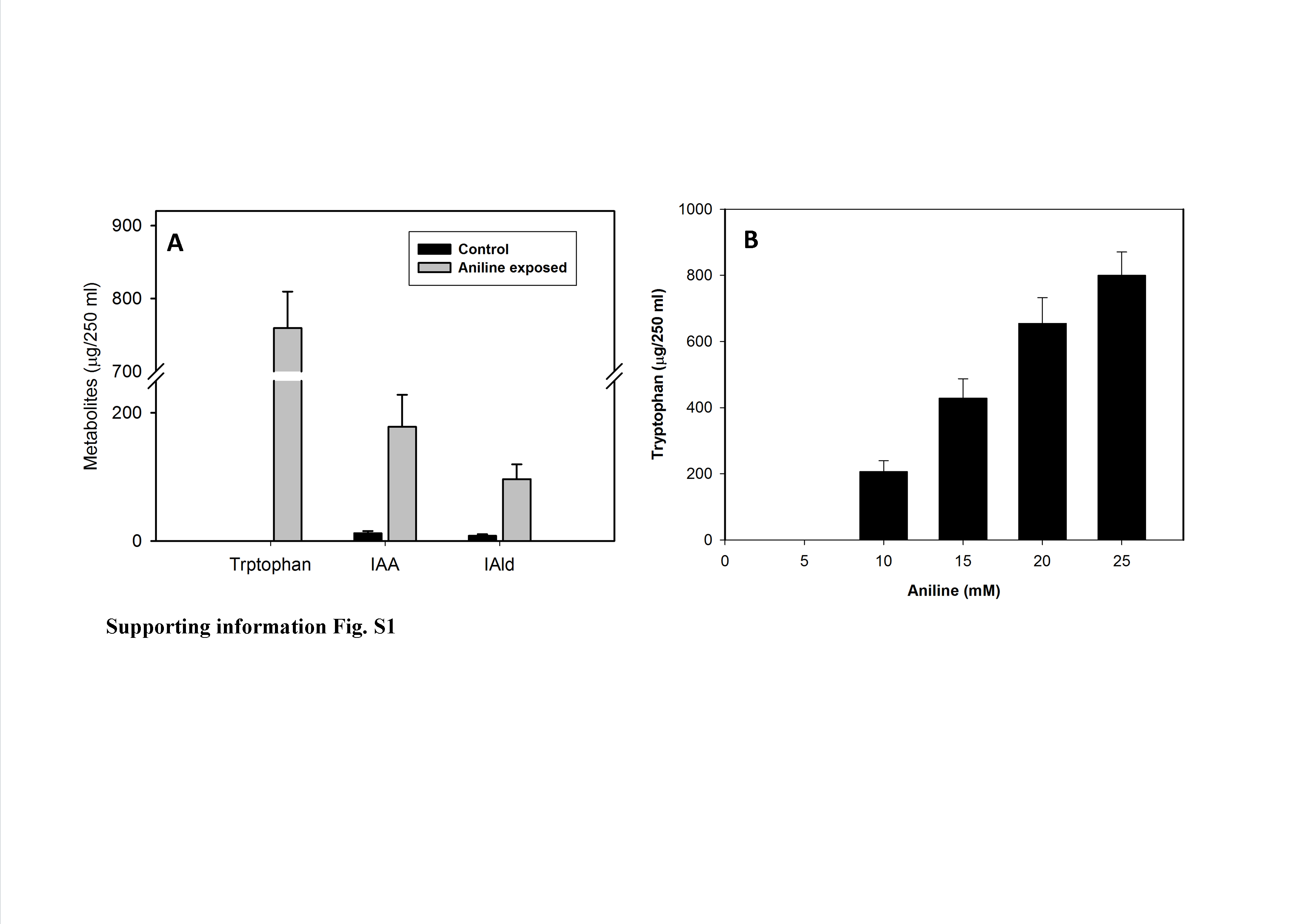

Supplement: Figure S1 — Tryptophan, IAA and IAld levels in control and aniline exposed cultures of R. benzoatilyticus JA2 (A). Tryptophan levels at different concentrations of aniline (B). Metabolites were quantified by HPLC and data represents mean standard deviation of three independent experiments. (TIF) [file pone.0087503.s001.tif]

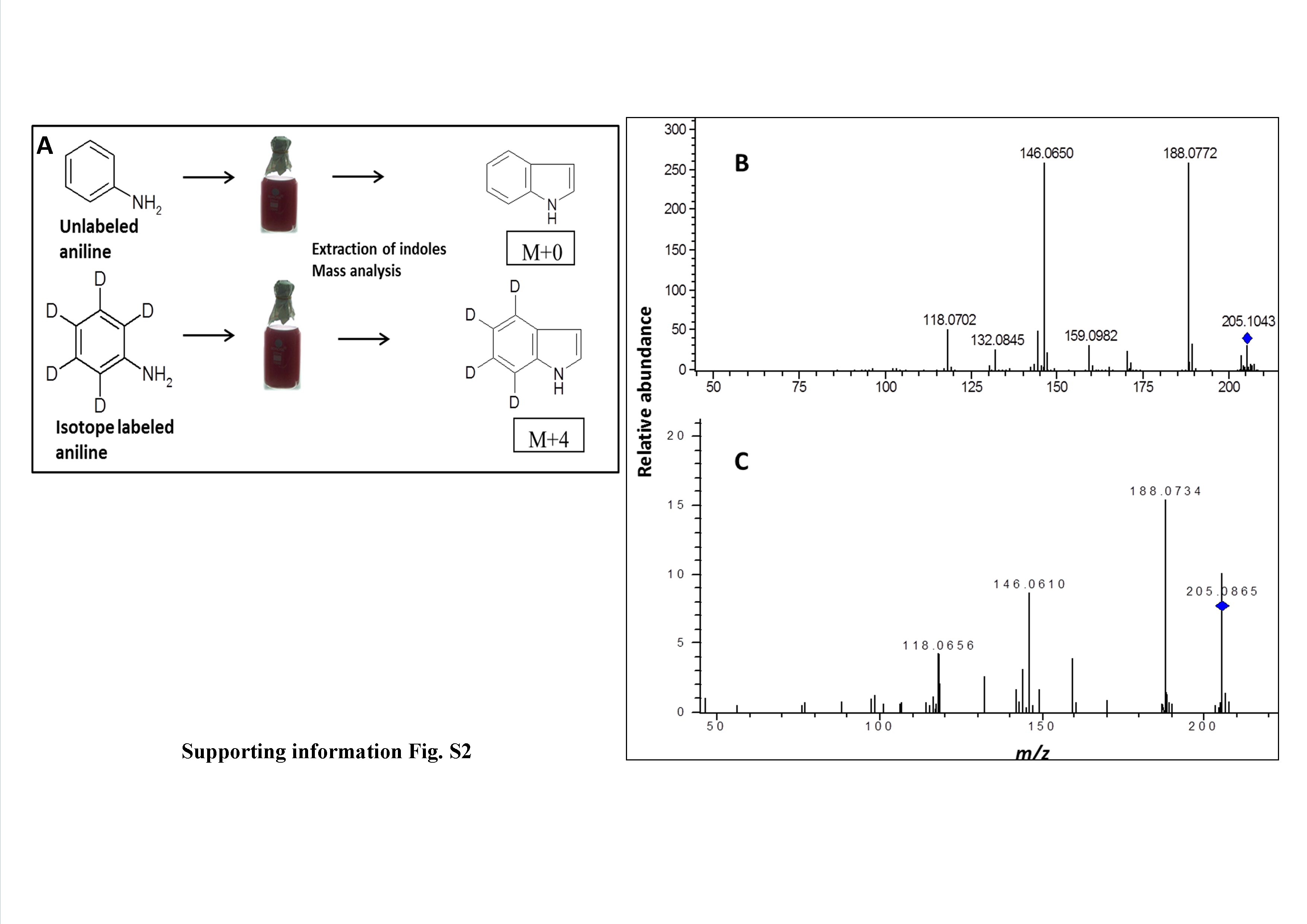

Supplement: Figure S2 — Schematic representation of stable isotope labelled aniline precursor feeding experiments with R. benzoatilyticus JA2 (A). M, denotes molecular ion mass and 0, 4 number of deuterium atoms incorporation. Mass spectrum of tryptophan from unlabeled fraction (B) and mass spectrum from labeled fraction (C). (TIF) [file pone.0087503.s002.tif]

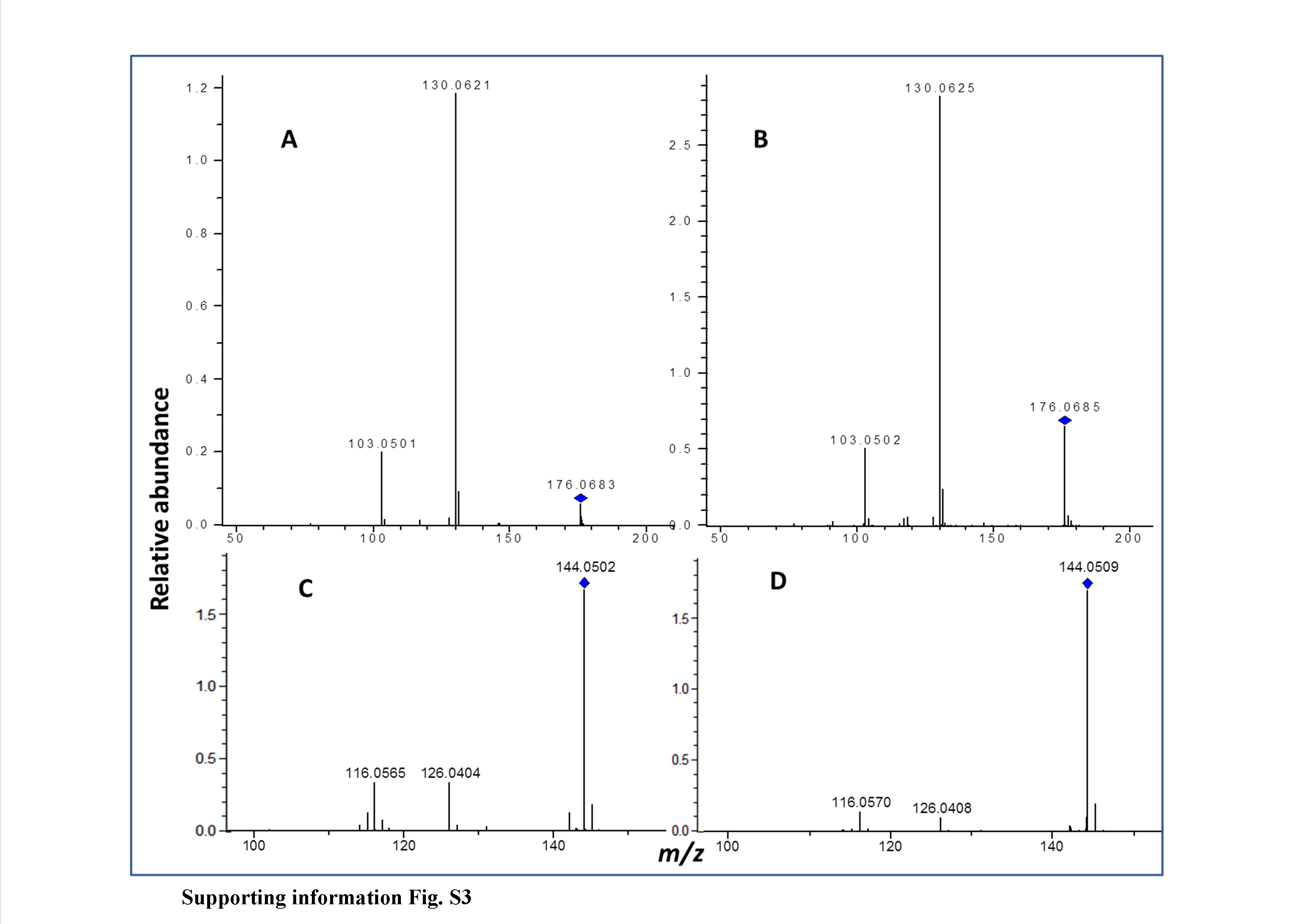

Supplement: Figure S3 — Mass spectrum of IAA from unlabeled (A) fraction and labeled fraction (B). Mass spectrum of IAld from unlabeled fraction (C) and from labeled fraction (D). (TIF) [file pone.0087503.s003.tif]

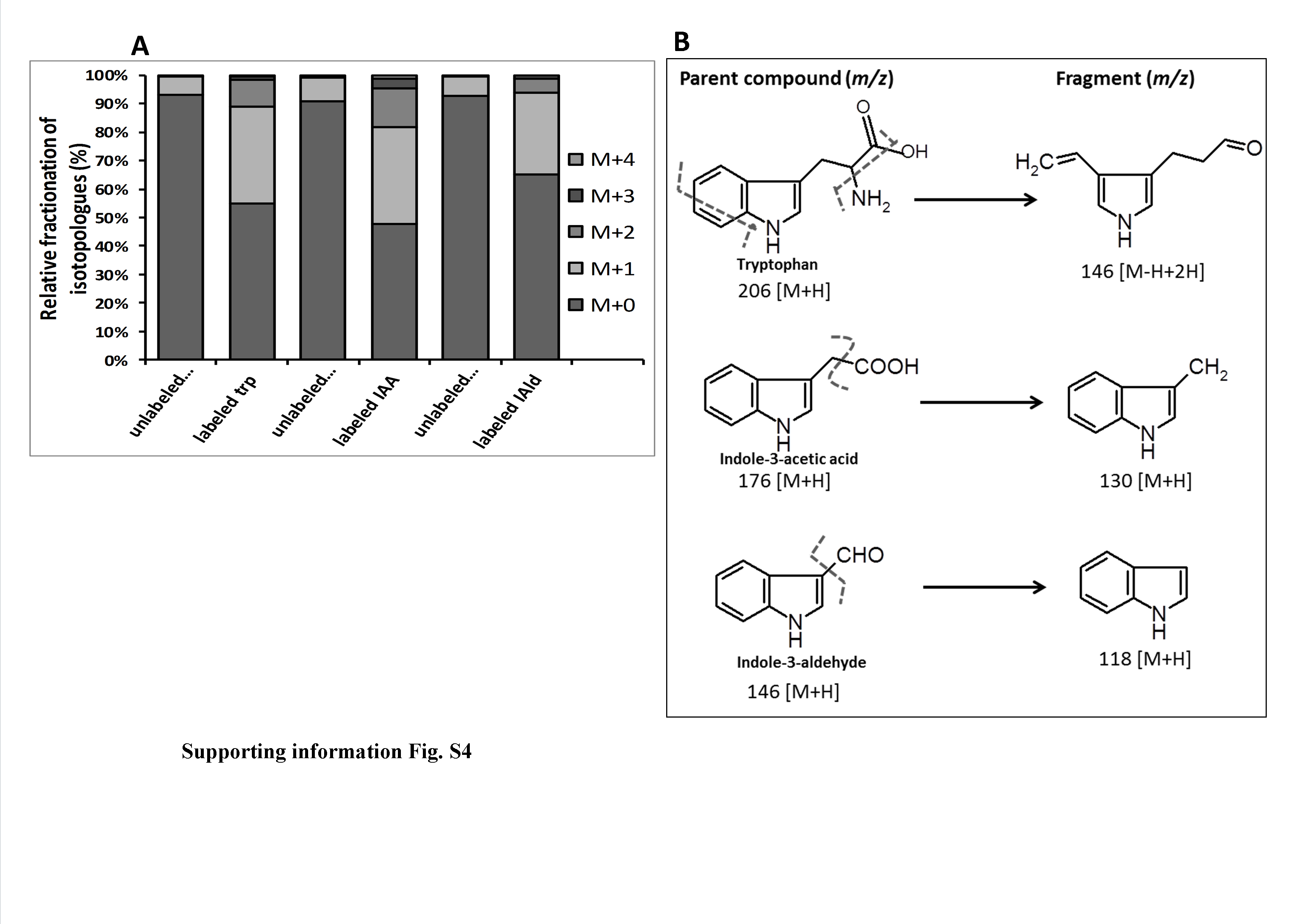

Supplement: Figure S4 — Relative abundance of isotopologues of indoles metabolites from unlabeled and labeled fractions. Area of each isotopologue obtained from mass analysis was used for the analysis (A). Predicted mass fragmentation of tryptophan, IAA and IAld according to Metlin data base (www.metlin.scripps.edu] (B). (TIF) [file pone.0087503.s004.tif]
