# Supplementary material for: Aniline Is an Inducer, and Not a Precursor, for Indole Derivatives in Rubrivivax benzoatilyticus JA2
Source: PLoS One. 2014 Feb 12;9(2):e87503. doi: 10.1371/journal.pone.0087503 (PMC3922755; doi:10.1371/journal.pone.0087503)
Supplement: Table S1 — Primers used for real time PCR analysis. (DOC) [file pone.0087503.s005.doc]

**Table S1** Primers used for real time PCR analysis.

| ***Gene Name* (Accession number)** | ***Sequence*** |
| --- | --- |
| Phospho-2-dehydro-3-deoxyheptonatealdolase (gi|332525529) | FP 5'-AGTACCTGGCCGACCTCAT-3'  RP 5'-CGTCGGTGCCGTTCTTGAA-3' |
| Phospho-2-dehydro-3-deoxyheptonatealdolase (gi|332525163) | FP 5'-ATGTTGCCGTCGGTGCCG-3'  RP 5'-GTGATCTCGCCGCAGTACA-3' |
| Chorismatemutase (gi|332527557) | FP 5'-GTTCTTCACCGACAGCACG-3'  RP 5'-CGAGTTCGGCCTGCACGT-3' |
| Anthranilate synthase (gi|332524751) | FP 5'-TGGCCGATGACGACCTGC-3'  RP 5'-GCTGCGTTATTCGGTCACC-3' |
| Tryptophan synthase_beta(gi|332524263) | FP 5'-CCGTTGTCGTCCTGCAGC-3'  RP 5'-GCAGCAACGCGATGGGCA-3' |
| Chorismate synthase (gi|332526664) | FP 5'-CAAGTGGCTGAAGGAACGC-3'  RP 5'-GTGTTGGCGGCGAAGAAGT-3' |
| 16s rRNA(gi|343201659) FP 5'ACCTGAAGAATAAGCACCGG3' | FP 5'ACCTGAAGAATAAGCACCGG3'  RP5'-AATGCAGTTCCCAGGTAAGC-3' |
