# Supplementary material for: Aniline Is an Inducer, and Not a Precursor, for Indole Derivatives in Rubrivivax benzoatilyticus JA2
Source: PLoS One. 2014 Feb 12;9(2):e87503. doi: 10.1371/journal.pone.0087503 (PMC3922755; doi:10.1371/journal.pone.0087503)
Supplement: Table S2 — Mass spectral analysis of Trp, IAA and IAld from stable isotope labeled fumarate fed cultures. M denotes molecular ion mass; 1,2,3,4 denote number of deuterium atoms incorporated. NF, no fragmentation was obtained as they were in trace amount (<1% abundance). Molecular ion masses in bold indicate no deuterium atoms incorporation. (DOC) [file pone.0087503.s006.doc]

**Table S2** Mass spectral analysis of Trp , IAA and IAld from stable isotope labelled fumarate

fed cultures

| **Compound** | **Exact mass** | **Observed mass**  **(*m/z*)** | **Fragmentation (%intensity)** |
| --- | --- | --- | --- |
| Tryptophan | 204.23 | **205.093 [M]^+^** | **205 (50), 188 (100), 146 (60)** |
|  |  | 206.068 [M+1] | 206 (16.6),189 (100), 147(8.3) |
|  |  | 207.108 [M+2]^+^ | 207 (100), 190 (70), 148 (38) |
|  |  | 208.110 [M+3] ^+^ | 208 (40), 191 (30), 149 (18) |
|  |  | 209.101 [M+4] ^+^ | NF |
| Indole-3-acetic acid | 175.18 | **176.143 [M]^+^** | **176 (100), 130 (66.6)** |
|  |  | 177.048 [M+1] | 177 (100), 131 (15) |
|  |  | 178.082 [M+2]^+^ | 178 (100), 132 (50) |
|  |  | 178.988 [M+3] | 178.9 (100), 132.98 (43 |
|  |  | 180.088 [M+4] | NF |
| Indole-3-aldehyde | 145.16 | **146.062 [M]^+^** | **146 (87.8), 118 (100)** |
|  |  | 147.092 [M+1]^+^ | 147 (40.9), 119 (100) |
|  |  | 148.104 [M+2]^+^ | 148 (42.8), 120 (100) |
|  |  | 149.020 [M+3]^+^ | 149 (100), 121 (17.5) |
|  |  | 150.050 [M+4]^+^ | NF |

M denotes molecular ion mass; 1,2,3,4 denote number of deuterium atoms incorporated. NF, no fragmentation was obtained as they were in trace amount (< 1% abundance). Molecular ion masses in bold indicate no deuterium atoms incorporation.
